# Possibilities of Increasing Effectiveness of RC Structure Strengthening with FRP Materials

**DOI:** 10.3390/ma14061387

**Published:** 2021-03-12

**Authors:** Wit Derkowski, Rafał Walczak

**Affiliations:** 1 Department for Building Technology, Linnaeus University, SE-351 95 Växjö, Sweden; 2 Chair for RC and RC Structures, Faculty of Civil Engineering, Cracow University of Technology, 31-155 Cracow, Poland; rafal.walczak@pk.edu.pl

**Keywords:** FRP, adhesives, material properties, strengthening, anchorages, galvanic corrosion

## Abstract

Modern composite materials based on non-metallic continuous fibres are increasingly used in civil engineering to strengthen building structures. In the strengthening of reinforced concrete (RC) structures, the utilisation of externally bonded fibre-reinforced polymer (FRP) composites is only up to 35% because of the pilling-off failure mechanism. This problem can be solved using pre-tensioned composite laminates. Due to more complex behaviour, the strengthening of structures by means of prestressing technology needs a careful design approach and a full understanding of the behaviour of both the materials and elements. The advantages and risks of the presented technology, which may determine the success of the entire project, will be highlighted in the paper. The possibility of using a flexible adhesive layer in carbon fibre reinforced polymer (CFRP) strengthening applications for flexural strengthening of RC elements, as an innovative solution in civil engineering, will also be presented. Parallel introduction of the flexible adhesive layer (made of polyurethane masses) and a traditional epoxy adhesive layer in one strengthening system was investigated in the laboratory tests. This solution was used for the repair and protection of a previously damaged RC beam against brittle failure.

## 1. Introduction

The issue of the sustainability, durability, and safety of a building structure is important at each stage of its life cycle. It is important to emphasise the role of these aspects during the planning, design, and execution of repairs and strengthening of existing civil and engineering structures. Since corrosion is one of the main factors affecting the durability of concrete building structures, especially those exposed to harmful environmental influences, for many years there has been a desire to find material and construction solutions that are resistant to corrosion and at the same time easy to use. When these solutions appear, they often become more widely used without deeper understanding.

In the 1980s, fibre-reinforced polymers (FRPs) based on non-metallic continuous fibres were first applied in civil engineering. Already since then, intensive scientific research began, recognising the advantages and disadvantages of this material—pioneering experimental research in this field was conducted by U. Meier at Empa—Swiss Federal Laboratories for Materials Science and Technology [1]. Shortly thereafter, numerous tests were carried out in many laboratories all over the world [2,3,4,5,6,7,8], e.g., at the Technical University of Lodz in Poland [9].

In general, strengthening with FRP composites significantly contributed to improving the flexural and shear capacity and increasing the fatigue life of structural members [10]. Practically until now, efforts are being made to avoid premature peeling off products made of this outstanding but also expensive material [11], e.g., externally bonded reinforcement on grooves (EBROG) and externally bonded reinforcement in grooves (EBRIG) bonding technique [12,13] or mechanical anchorage application [14]—these solutions allowed for some improvement in the efficiency of externally bonded FRPs.

The use of the FRP composites for prestressing building structures has been started from the mid-1990s of the 20th century. Initially, only prestressing tendons made of carbon or aramid fibres were applied for structural strengthening, and after a few years, carbon fibre reinforced polymer (CFRP) strips have also been used for prestressing. Nowadays, on the market, there are several, significantly differing, anchoring systems for the pre-tensioned CFRP laminates which allowed to significantly increase the utilisation of the material used. This technology of strengthening was first applied in Gomadingen, Germany, in October 1996 for Lauter Bridge [15], and the first application in Poland of strengthening of a large-area roof of an existing industrial building by means of prestressing with CFRP strips appeared in 2006 [16].

The use of a flexible adhesive layer in CFRP applications for flexural strengthening of RC elements is an innovative solution for the safety improvement of the repaired structure [17,18,19,20]. Parallel use of the flexible adhesive layer (made of very low stiffness polyurethane masses) and a typical epoxy resin adhesive in one strengthening system seems to be the desired solution, especially for structures that have to be protected against the brittle failure arising, e.g., as a result of sudden peeling off of the FRP material.

## 2. Summary of Characteristics of FRP Materials

Materials based on carbon, aramid, glass, or basalt fibres are innovative construction composites and they are increasingly used in civil engineering nowadays. The ongoing effort is to find other cheaper, more easily available, and, at the same time, durable and sufficiently robust types of fibres that can be used in FRPs used in civil engineering. Increasingly favourable are organic natural fibres such as bamboo, flax, hemp, jute, sisal, and coir fibres [21].

The majority of those fibres have a tensile strength noticeably greater than the strength of reinforcing steel. Since they are non-metallic fibres, they can be considered as high resistance to corrosion material. Their density is more than a few times smaller compared to steel. Moreover, glass and carbon fibres are resistant to UV rays, while aramid fibres have some interaction with this radiation [22]. On the other hand, most types of glass fibres are not resistant to an alkaline environment which can be a problem when applied directly to concrete. 

Matrix in composites is usually made of epoxy resins, sometimes polyester and vinyl ester resins. The resistance of resins to the impact of chloride ions is very good, making that the FRP composites are resistant to de-icing salts. FRP materials are created by embedding a very large number of non-metallic fibres in a matrix which allows for even distribution of the tensile force on all the fibres. It has virtually no influence on the tensile strength of the FRP material, whereas it determines the shear and compression capacity of the composite. In addition, the matrix protects the fibres from mechanical damage and unfavourable environmental influences. Comparison of the estimated properties of most popular composite materials made on glass fibres (GFRP), basalt fibres (BFRP), aramid fibres (AFRP), or carbon fibres (CFRP), compiled from a variety of manufacturer’s catalogues and literature sources [22,23,24,25], is shown in Table 1.

Laminates in the form of long, straight portions of the strips are obtained by pultrusion. For this purpose, collimated continuous fibres are pulled through the hot fluidised resin and then after removal of the excess of resin pass through the forming mould. Then, under appropriate temperature and pressure conditions, the matrix is cured [22].

The time-dependent properties of composite materials have an important influence on the durability of structural strengthening, particularly in the case of pre-tensioned laminates. The change in the FRP composite behaviour over time is a result of the rheological properties of each of the constituent materials and the changes occurring in the contact zone between these materials (adhesive bonding). Table 2 shows the rheological properties of the most commonly used composite materials given by Borosnyói and Balázs [26]. The approximate values of the long-term tensile strength, taking into account the rheological phenomena occurring in a period of 100 years, are also shown.

The most common adhesives used for FRP applications are materials based on the same resins that were used as FRP composite matrices. Epoxy resin adhesives, which are the most widely used, are relatively stiff. They have high shear and tensile strength (up to 30 MPa), but they are characterised by a low range of ultimate strain (usually under 4%), and thus are inappropriate in applications in which high deformability exists.

An alternative to the use of epoxy resins is using polymeric flexible adhesives. These materials made of polyurethane (PU) mass are elastomeric and have a tensile strength of only a few MPa but a gigantic deformation range. Adhesive bonds made of PU have a higher ductility and strain energy than epoxy resins, which can be measured by the area under the stress–strain curve [27]. The basic material properties of epoxy resins and polyurethane adhesives are shown in Table 3.

**Figure 1:**
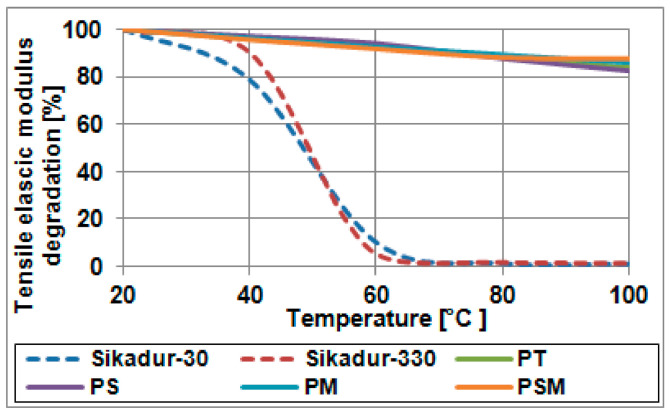
The tensile elastic modulus degradation in elevated temperatures (compare—Table 4).

Another difference is the behaviour of adhesives at elevated temperatures, which is often important in industrial facilities. Resin-based adhesives show a decrease in a tensile elastic modulus of about 90% already at 60 °C and a decrease of as much as 99% at 100 °C. On the other hand, polymer flexible adhesives at 100 °C show a decrease in a tensile elastic modulus only of about 17% for rigid polymers (PS, PT—see Table 3) and 13% for soft polymers (PM, PSM—see Table 3). This acknowledges the polyurethane adhesives can be used even at elevated temperatures. Table 4 and Figure 1 present the tensile elastic modulus change observed at various temperatures.

Polymers are viscoelastic materials, therefore their stress–strain curve is contingent on the speed at which the material is deformed, which is used to reduce dynamic actions in structures [29]. In tension, an increase in the deformation velocity increases the limit deformability of the PM polymer and its strength and stiffness. In compression, an increase in stiffness of the PM polymer is observed as the deformation speed increases.

The durability of building materials during their lifetime is one of the most important issues [30]. PM polymer was tested in this respect during the tests in the accelerated ageing chamber. Moreover, its behaviour was observed in conditions of natural exposure to environmental and chemical factors at the Balice Airport [31]. This material was also tested for 60 days using an accelerated ageing chamber in accordance with the American Society for Testing and Materials ASTM G154a standard, which allowed us to simulate the interaction of environmental factors for 3–5 years [32]. The optical comparison of PM polymer before and after ageing showed differences in appearance but the material degradation is superficial and is only significant for thin polymer layers exposed to environmental exposure, while it is insignificant for the bulk of the polymer. The tensile stress reduction factor after ageing was found as 0.75. At compression, the effect of ageing was small because the surface degradation affected a much larger section area.

In the case of high deformation under live loads (e.g., thermal), the flexible joint polymer significantly reduces the amount of stress under cyclic loads. This is the so-called Mullin effect, which is characterised by the reduction of stress in the polymer during the first several load cycles and caused by the reconstruction of the internal structure of the polymer. As with the relaxation phenomenon, the degree of stress reduction depends on the level of initial deformation during cyclic loading—the greater the strain, the greater the degree of stress reduction.

## 3. Increase in the Strengthening Effectiveness by Using Pre-Tensioned FRPs

Due to composites’ expedient properties, the high potential in pre-tensioned FRPs application is clearly visible. Moreover, high corrosion resistance, high strength, and stiffness-to-weight ratio, high energy absorption, and very good fatigue resistance are also highly important in structural strengthening. Pre-tensioned FRPs can control structural deterioration that occurs over time and sustain the impacts of vehicles much better than prestressing steel. Moreover, seismic upgrading and repurposing of the structure are easily conducted with these materials [10,33,34,35,36]. 

The application of pre-tensioned composite laminates enables the strengthening execution almost without the structure dimensions or self-weight change. However, the bearing capacity of bent or tensile members is significantly improved. Moreover, the serviceability limit states of the strengthened structure are improved by decreasing the deflection and cracks width (completely closing the cracks in some cases). Therefore, structure durability is enhanced. In the case of pre-tensioned laminates, preparation of the concrete surface is not crucial (in contrast to the passive FRP strengthening) since the prestressing force is transmitted mainly through the special anchorages. In addition, the unbonded pre-tensioned strips application is also feasible. Another advantage of this technique is the fact that FRPs are active reinforcement and can carry loads immediately after their pre-tensioning (without the necessity of occurrence of the further structure strains). Moreover, the degree of FRPs utilisation is much higher comparing to passive bonded strips.

Aramid fibre-based composites are very good under both static and dynamic loads. According to Deng and Xiao [37], AFRP proves to perform very well in strengthening structures subjected to cyclic loading by prestressing. It also possesses stronger protection against high temperature, corrosion, and adverse environmental effects compared to prestressing steel. However, AFRP has some less significant disadvantages—according to Kurihashi et al. [38] they have poorer behaviour in acidic and alkaline media, which CFRP materials can more effectively resist.

Glass fibre composites are unfortunately characterised by a relatively low modulus of elasticity, which makes the utilisation of GFRP material in the strengthening of reinforced concrete structures very limited. The knowledge about the possibility of strengthening building structures with initially pre-tensioned GFRP is still quite minimal. Research in this area was conducted by Lin et al. [39] and it has shown that pre-tensioned GFRP can be an effective solution for increasing the load-carrying capacity of reinforced concrete beams.

There is a significant decrease in both AFRP and GFRP in their tensile strength when subjected to prolonged constant loading, while many studies have shown that CFRP is much less sensitive under the same load conditions.

In general, pre-tensioned CFRPs are appropriate in cases in which strength, stiffness, lower weight, and fatigue are critical issues [40]. Based on Aslam et al. [41], the key benefits of post-tensioning with CFRP are its lightweight, extremely high tensile strength, high corrosion resistance, excellent rheological properties, electromagnetic neutrality, fast and simple construction, and low operating cost. It should also be mentioned that during carbon fibre production, there is an ability to modify the elastic modulus in a very wide range, which can be an advantage in many applications. Moreover, the application of hybrid FRP (e.g., carbon-glass) is feasible [42].

### 3.1. Prestressing Systems for CFRP Strips

The crucial aspect of strengthening reinforced structures with externally bonded FRP is the peeling-off mechanism. The concentration of shear and normal stresses follows at the ends of the laminate bonded to the concrete surface. As a result, material detachment occurs. To prevent this type of failure mechanism in the case of pre-tensioned FRP laminate, several methods of strip anchoring have been developed so far [43,44], which include both ideas with gradual reduction of prestressing force along the length of the strip and those using mechanical anchoring.

The following sub-sections describe several systems which enable structural strengthening by CFRP laminates prestressing.

#### 3.1.1. Anchoring System Using Specially Formed Polymer Heads

**Figure 2:**
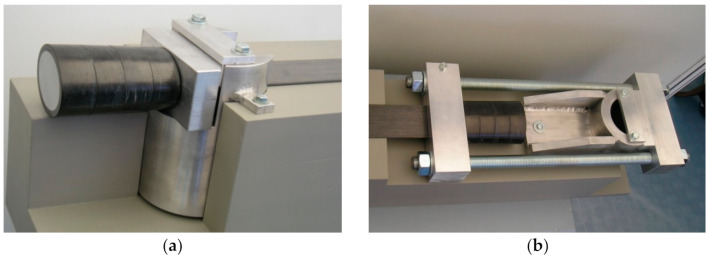
(**a**) StressHead passive anchorage and (**b**) StressHead active anchorage.

Schwegler and Breset propose a ‘StressHead’ system to enable anchoring strips prestressed up to the force of 220 kN each (laminate deformation of 9.5‰) [45]. A special reinforced polymer head is made at the end of the laminate. It has a length of 110 mm and an elliptical cross section 80 mm × 60 mm in axial dimensions. StressHeads are fixed on massive steel anchorages fastened to the reinforced concrete structure by Ø100 mm steel pin. Figure 2a shows a model of a passive (fixed) anchorage, and Figure 2b shows a model of an active (moveable) anchorage of the strip. Either adhesive bonded or unbonded strips are applicable in this prestressing system. 

The disadvantage of this technology is the requirement of prefabricated polymeric heads on both ends of the strip of designed length. Moreover, drilled deep holes with a large diameter are necessary to fix the anchorage. This may not be possible in the case of many strengthened structures. It should be noted, however, that the durability and corrosion resistance provided by this system is the same for the strip and the anchor head.

#### 3.1.2. Anchoring System Using Steel Clamping Jaws

In these systems, prestressed CFRP strips are fixed in the special steel jaw anchors. In addition, composite laminates are bonded to the strengthened structure over the entire length. Various types of these systems are available over the world (e.g., LEOBA, Polish systems SIKA and Research Institute of Roads and Bridges in Poland (IBDiM)).

LEOBA anchorage system, described by Andrä et al. [46], consists of an anchor plate fixed to the strengthened structure and the pressure jaw to anchor the pre-tensioned strip with the bolts. During the prestressing of the structure, additional jaws are provided to fix the strip end on the active anchorage side. The retaining block set directly behind the active anchorage is tightened to the steel anchor plate which is fixed in the structure. The main advantage of the LEOBA system is the possibility of using a very small-sized hydraulic device to pre-tension the strip.

SIKA and IBDiM system, developed by Łagoda [47], is a certain modification of the LEOBA based on the gradually tensioned and sequentially anchored strip. Four independent jaws form an anchorage to fix the strip. As a result, the prestressing force is gradually decreasing at the anchorage length.

**Figure 3:**
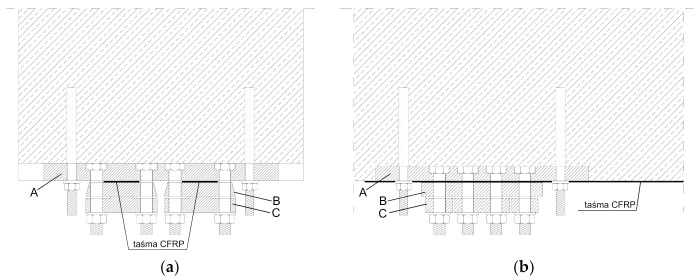
(**a**) Transverse and (**b**) longitudinal cross-section of fixed anchor for two tendons.

**Figure 4:**
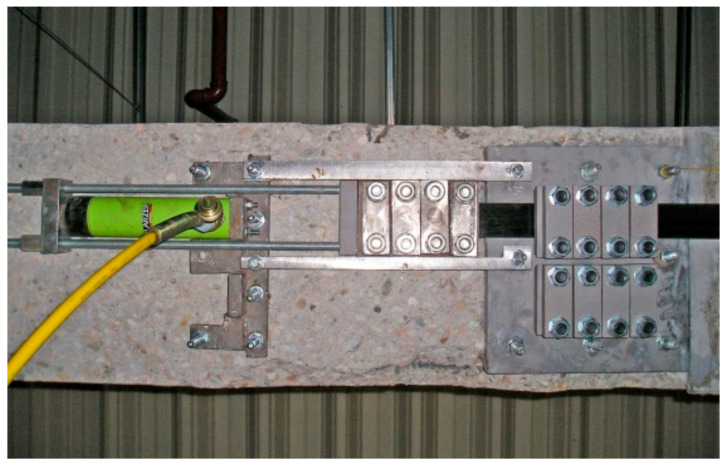
View of live anchorage.

A transverse and longitudinal cross-section of the anchorage designed for two CFRP tendons is shown in Figure 3 [48]. Laminates’ anchorage is realised by their pressing with the blocks ‘C’ through the intermediate plate ‘B’ to anchorage basement ‘A’. Elements ‘C’ are appropriately shaped in order to evoke the uniform pressure distribution along the whole length of the tape. Pressure is produced by M16 bolts screwing with a dynamometric key. Each tendon is gradually tensioned and fixed in sequences (in four stages) to the live anchorage from the active side and to the fixed anchorage from the passive side. After the implementation of the required value of prestressing force, the tendon is finally mounted from the active side to the fixed anchorage and tensioning jaws (live anchorage) are dismounted. Disassembly of tensioning jaws took place in a specified sequence to reduce gradually the tape pressure. A view of live anchorage during the tensioning process for the first tendon is presented in Figure 4.

#### 3.1.3. Anchoring System of Screw Fixing Prefabricated Tendon to the Element

**Figure 5:**
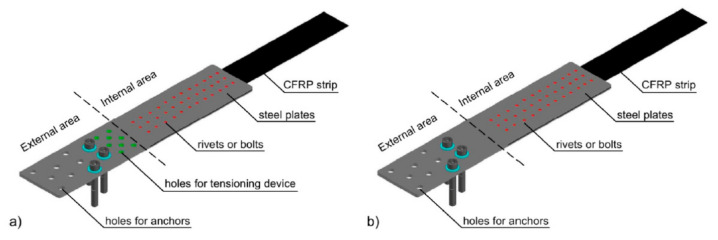
The NEOXE anchoring system: (**a**) active side and (**b**) passive side [50].

An example of this type of solution is the NEOXE Company (Warsaw, Poland) anchorage system, in which the composite laminate is anchored in prefabricated components, according to Siwowski et al. [49,50]. The composite is glued between steel sheets using epoxy resin. Moreover, to enhance the adhesive layer, metal mechanical fasteners connect the laminate to the anchor plates. There are two types of anchorages—N-type with hybrid bonded/riveted joints and S-type with bonded/bolted joints. In this system, the CFRP strip with determined length is delivered on-site as ready-to-install, i.e., with two assembled steel anchorages (active and passive one) on each strip ends—see Figure 5. 

#### 3.1.4. Non-Mechanical Anchorage Systems

The world’s first system for prestressing CFRP strips without mechanical anchorages was developed in EMPA Research Laboratory in Zurich, according to Kotynia et al. [51]. The prestressing force is transferred from the strips to the structure only by the adhesive layer made of epoxy resin. A gradual decrease in the prestressing force over the strip’s length is provided to reduce the shear stress at the composite ends. The prestressing force in strengthened regions varies from the maximum value in the half-length of the strip to zero at the ends of the strip. As a result, delamination of the strip’s end is prevented.

Swedish Tenroc Technologies company developed another technique of the application of pre-tensioned CFRP strips with non-mechanical anchors. The system is based on a multi-segment tensioning device to apply gradually increased prestressing force.

To speed up the application of those systems, accelerated curing of the adhesive layer can be achieved through heating. Electro-conductive properties of the carbon fibres embedded in the CFRP strip can be utilised as a result of the flow of electrical current in the strip and the temperature of the composite, and then the epoxy adhesive is significantly increased. Therefore, the curing time of the epoxy adhesive may be reduced using this technology from several days to about two hours.

### 3.2. Benefits and Drawbacks of Using FRP Prestressing Systems

Although the number of tests on structures strengthened with pre-tensioned CFRP strips (Kałuża and Ajdukiewicz [52], Young-Chan et al. [53], Kotynia et al. [54]) is much smaller than those performed for passive strengthening (externally bonded non-stressed FRP composites), it can already be stated that the developed laminate anchoring technologies give a very satisfactory effect. Numerous tests have shown that 100% utilisation of the FRP material is possible and that the increase of the obtained ultimate load-bearing capacity exceeds 150%.

Observing the work of the FRP material, it is important to highlight much bigger elastic elongation than conventionally used prestressing steel (~0.2% for steel compared to ~1.6% for CFRP). Due to that circumstance, in the tensioned steel tendon exposed to wedges slippage in the anchorage (or other similar effects), which decreases its initial elongation for few millimetres, a significant percentage of prestressing force is lost. However, in the composite tendon loss of prestressing force would be smaller (because the FRPs have a smaller modulus of elasticity than steel). This is also extremely important in conditions where it is necessary to consider the accidental loading, e.g., in structures that exist in seismic areas or those subjected to impact loads. When CFRP strips with an initial elongation of 0.9% (which could be equivalent to force 220 kN; this is the same as in monostrand steel tendon) are used, the prestressing system acts such as ‘a rubber band’ and each change in strain is within the elastic range of the material behaviour [55]. Thus, even with large cyclic strain changes on the composite tendon, the structure can still safely handle the load. On the other hand, in the case of a steel tendon it could already become yielded (resulting in irreversible deformations), and the strengthened structure can be damaged.

In addition to the great difficulty in the proper implementation of the thrust and anchoring of FRP tendons (which are very sensitive to even small eccentricities and local stress concentrations), one of the major disadvantages of this technology may be galvanic corrosion that occurs when two different materials are in direct electrical contact. The mechanism of galvanic corrosion of the anode steel, when coupled with the CFRP cathode, was studied in [56,57,58] and explained in [55].

Corrosion prevention in composite strips’ anchorages can involve non-conductive, suitable separation of the FRP laminates from the steel components. The easiest way would be to use epoxy resin as an adhesive for the CFRP strengthening, but according to Tavakkolizadeh and Saadatmanesh [56], this material does not provide appropriate durability because it may not guarantee a needed water-tightness, especially in case of prolonged exposure to salt solutions. Another problem is that in many anchorage systems, as a result of the mechanical joining of the CFRP strips (by means of bolts, screws, or rivets), the resin coating covering the carbon fibres can be locally damaged, thus establishing physical contact of the carbon fibre to the steel. This danger is particularly apparent in the solution of the NEOXE anchoring system, in which the mechanical fasteners are going through the CFRP laminate (see Figure 5). An intermediate layer of GFRP sheet can also serve as a baffle between the steel and CFRP layer but, according to Hollaway and Cadei [59], this additional layer is very difficult to use from the practical point of view.

## 4. Improvement of Structure Safety by Using Flexible Adhesives

**Figure 6:**
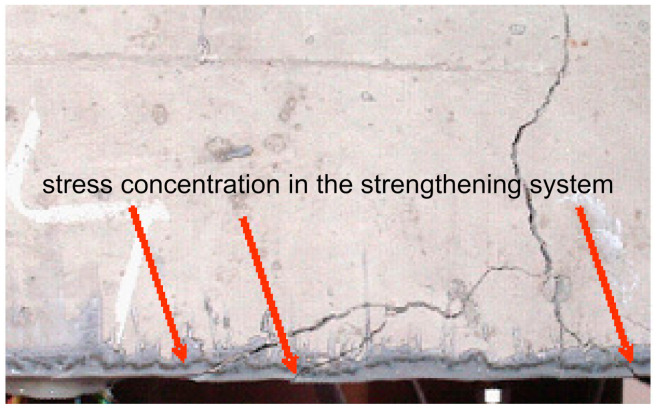
Local damages in a beam strengthened with carbon fibre reinforced polymer (CFRP) laminate bonded on epoxy resin adhesive generating high-stress concentrations in the strengthening system [17].

Repair and/or strengthening damaged concrete elements using FRP composites while ensuring simultaneously enough safety margin of the strengthened structure is a difficult task. When the structure is strengthened by CFRP laminates bonded on stiff adhesive layers made of epoxy resins, the advantages of CFRP materials are not fully utilised because of the low tensile and shear surface strength of the concrete element’s surface. Shear and normal interfacial stress peaks in the stiff bond system (concrete–epoxy) occur at the end of CFRP laminates or in discontinuous areas such as the crack regions. Local stress concentrations in the adhesive layer, and subsequently in the FRP laminate, are arising at the locations of cracks in the concrete element. In the case of rigid, relatively brittle, epoxy adhesives, even small stress concentrations can cause cracking, while soft polymeric adhesives have the ability to redistribute these stress concentrations and bridge the crack. The notch effect that appears after cracking of the RC element in both the adhesive layer and laminate is shown in Figure 6.

The possibility of using a flexible adhesive layer in CFRP strengthening applications for flexural strengthening of reinforced concrete elements as an innovative solution in civil engineering is presented in this section. It is obvious that regardless of the type of adhesive layer (hard epoxy resins or soft polymers) the failure of the strengthening system along the concrete surface can be initiated by the surface micro-damages (micro-cracks and cavities in the concrete structure). Therefore, proper preliminary preparation of the concrete surface always plays a fundamental role when using this type of strengthening. The parallel introduction of a flexible adhesive layer (made of polyurethane masses) and a traditional epoxy adhesive layer in one strengthening system was investigated in the presented research. This is one of the applications of the flexible joint method, which is developed at the Cracow University of Technology, uses PU polymers as adhesives in bonding FRP composites to concrete and masonry substrates and is registered in the Polish Patent Department as No. P-368173. The flexibility of the adhesive layer significantly increases the ductility of the structure. This solution was used for the repair and strengthening of an RC beam, severely damaged by fatigue load. The objective of this study was to explain the advantages of polymer flexible adhesives as the system fixing FRP composites to structural elements.

### Test of Parallel Use of Stiff and Flexible Adhesives for Increased Safety of Strengthened Structure

The object of the test was a beam damaged as a result of fatigue load and repaired in order to ensure temporary safety of use (e.g., until a new structure is made).

**Figure 7:**
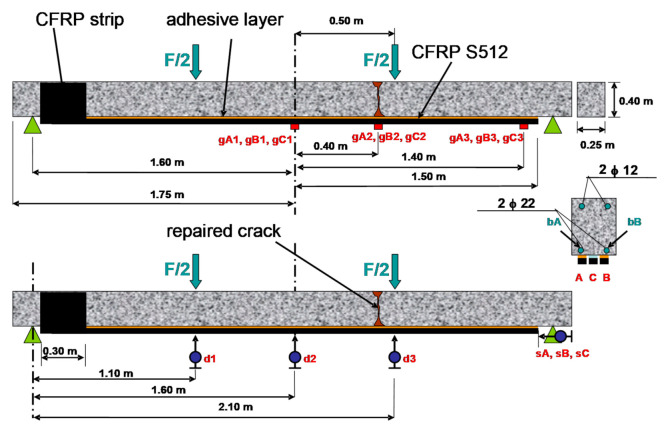
The test setup.

The tested RC beam (Figure 7) was originally made of the concrete C35/45 grade and reinforced with two Ø 22 mm bars of the steel class AII (design yield strength f_yd_ = 310 MPa) at the bottom and two Ø 12 mm of the steel class AIIIN (f_yd_ = 420 MPa) at the top. The beam was damaged after 696273 cycles of load (2 Hz frequency, σ_max_ = 0.7 f_y_, stress ratio R = 0.2). The bottom reinforcing bar ‘bA’ has failed in brittle fatigue form which caused that the ‘bB’ bar, which was partly cracked in fatigue, yielded, and the compression zone was crushed. Once the cyclic load has stopped and only a small permanent load has been left, the ‘bB’ rebar protected the whole failure beam against collapse.

**Figure 8:**
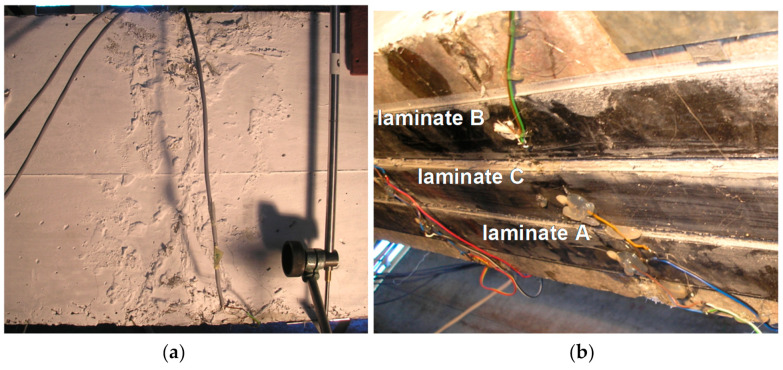
The repaired beam in the main breakage region: (**a**) side view and (**b**) bottom view.

The main breakage (crack pattern) of the beam and the crushed compression zone were repaired by injection using epoxy resin Sikadur 52 (Figure 8a). Next, the beam was strengthened by three CFRP S512 laminates at the bottom surface of the beam. Two of them, outermost, were bonded on the stiff adhesive layer made of epoxy resin Sikadur 30 (compare Table 3). Those laminates are presented as laminates A and B in Figure 8b (and in further analysis marked as A/S30 and B/S30). The third CFRP laminate (inner) was bonded on the very flexible adhesive layer made of very soft polymer, named PXBM (laminate C in Figure 8b, marked also as C/PXBM). The mechanical properties of the adhesive layer made of polymer PXBM are tensile elastic modulus E = 0.3 MPa and ultimate strain ε = 1000%. In order to protect the one end of CFRP laminates against delaminating, the band made of CFRP sheet was installed on one side of the beam.

Before the main damage test, the repaired beam was tested in few levels of static and cyclic loading. The first group consisted of three monotonic load cycles up to the force of 120 kN. In the fourth primary test, the beam was investigated in 11,000 cycles of load in the force range 30–110 kN with a frequency of 2 Hz. During this test, no damage was observed.

**Figure 9:**
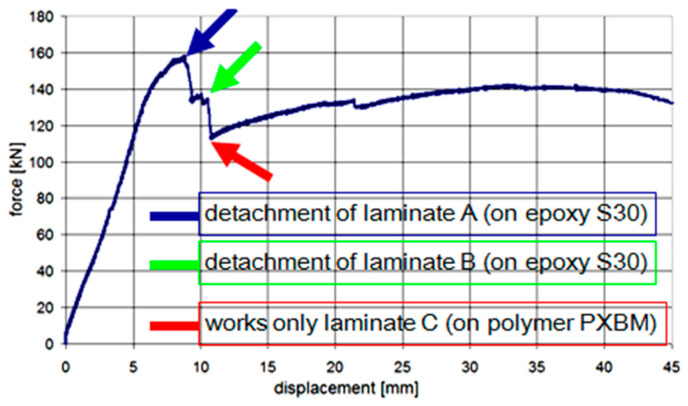
The force-displacement (F–d2) diagram presenting steps of the beam work.

After these tests, the main static test up to the failure was carried out with the displacement rate of 1 mm/min (displacement of the actuator jack). In the first phase of load, all CFRP laminates worked up to the maximum external force of 158.1 kN when the first of the side laminates (marked as A) was detached. Consequently, eccentric distribution of internal forces occurred and soon afterwards the second side laminate (B) also detached. The obtained relationship force–displacement is presented in Figure 9.

**Figure 10:**
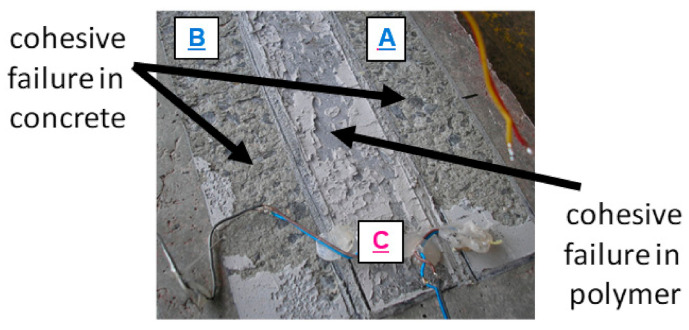
The bottom of the tested beam after the laminate detachment: side laminates (A and B)—brittle failure in concrete, middle laminate (C)—ductile failure in the adhesive layer.

The main effort (the biggest strain) of laminates was observed in the place of repaired crack, and the notch effect in this place initiated delamination of the laminate A in the failure place of the bar ‘bA’ (visible as the force drop to the value of 132.6 kN—Figure 9). Just after that, rapid detachment of the laminate B appeared in the identical form as the laminate A (visible as the force drop to the value of 112.9 kN—Figure 9). Detachment occurred in the concrete cover (Figure 10). Since that moment, the beam capacity was provided only by the laminate C, which was bonded on PXBM—an extremely soft polymer.

**Figure 11:**
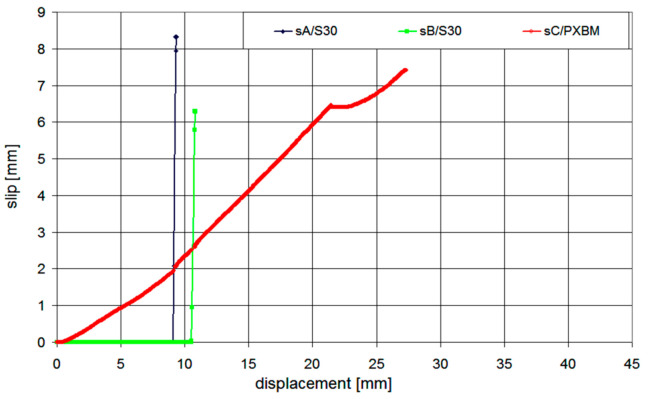
The laminate end slips, measured at the points sA, sB, and sC.

**Figure 12:**
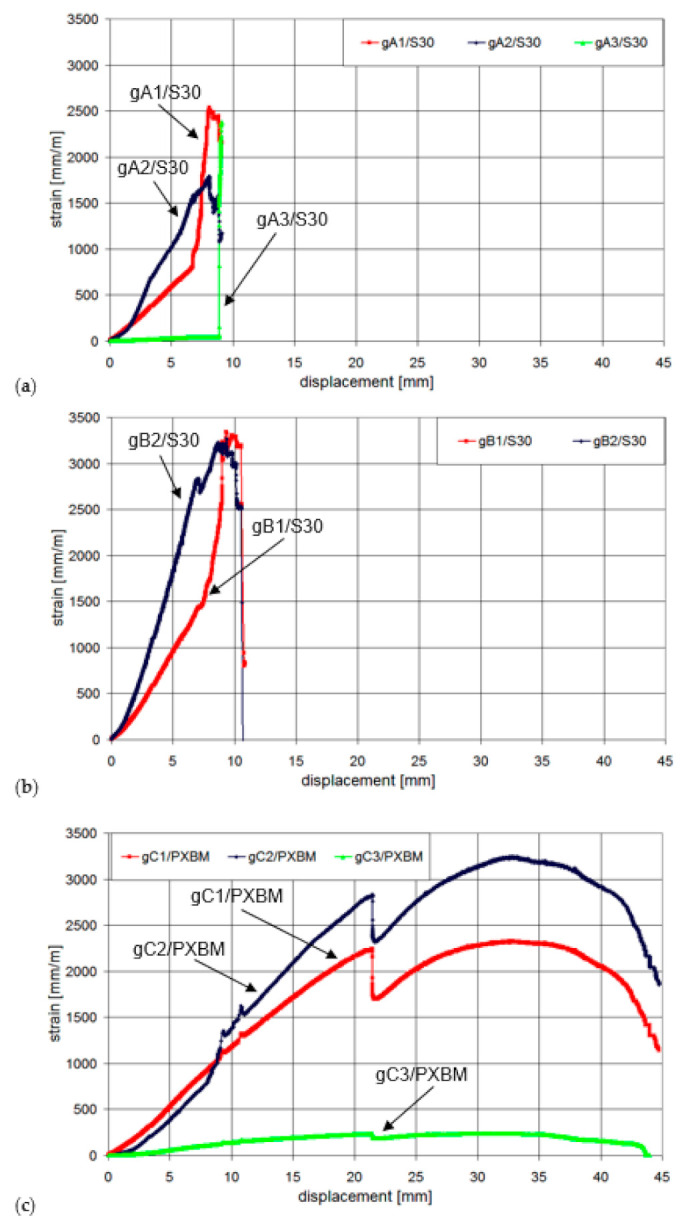
Changes of the laminate strains at the points (**a**) A/S30; (**b**) B/S30, and (**b**) C/PXBM.

The calculated ratio between the deformation energy value (calculated as an area under the force–displacement graph) obtained after the detachment of the laminates A and B (5120 J) and the work value up to the failure of the laminate A (823 J) is 6.22. This high deformation energy reserve in the post-critical state gives a high increase in structural safety. The laminates A and B were not showing any slip of their ends up to the laminates’ detachment, whereas the laminate C showed the slip of value 2 mm just before the failure moment of the laminate A—see Figure 11. The slip increased only up to the value of 2.8 mm and the slip gradient increase of the laminate C started to be higher, when the laminate B detached. The laminate C was able to carry the load up to the force of 142 kN, and next, a very slow process of delamination started in the polymer adhesive layer, with almost constant force and without damage of concrete—see Figure 10. The end of the C plot at the value of 7.5 mm (Figure 11) was caused by the limit of the linear variable displacement transducer LVDT sensor. Even partial crushing of the compression concrete zone (at displacement equal 22 mm) did not cause any significant force to drop and significant deflection increase of the beam (Figure 9). It caused only a temporary pause of the slip increase (Figure 11) and temporary reduction of strain in the laminate C (Figure 12—the strain history at points gA2, gB2, and gC2), because of stress redistribution.

Insensitiveness to the notch effect of the laminate C and ability to the absorption of deformation energy by the polymer adhesive layer (observed in such advanced stadium of damage process) stem from the high flexibility of the polymer PXBM [60]. The laminate C works properly in the initial phase of damage of the polymer layer when the beam deflection was equal to 37 mm.

## 5. Discussion and Conclusions

Threats to the durability of structural components made of steel exposed to unfavourable environmental conditions have resulted in a growing interest in the use of modern composites based on non-metallic materials. Considering the numerous advantages of FRPs, including their very low weight and the possibility of manufacturing practically any length of units, and thus also the ease of installation, many designers are beginning to consider these materials as a substitute for traditionally used steel reinforcement.

The innovative technology of pre-tensioned FRP laminates allows the entire use of material properties and is more and more popular, especially in the case of bridges or industrial structures strengthening. The cost analysis of the project must consider the price of the material used and the cost of the construction work (equipment, working time), which in this case may be much lower. It is also important to consider the possible reduction of a number of social costs resulting from the reduction of the time needed for the execution of a strengthening—for example, during the rehabilitation of a road viaduct the time needed to take the traffic route out of use may be significantly reduced if not completely eliminated. However, it cannot be said nowadays that the technology of reinforced concrete structures with externally bonded composites is fully developed and, in each case, it will be the best solution. A number of implementation problems arise from the fact that FRP composites are materials working practically uniaxially, i.e., they show negligible strength in the direction perpendicular to the fibres and virtually do not bear bending moments—this is why even small eccentricities at the stage of strip tensioning cause premature failure of the laminate. The issue of special fire protection of external reinforcement is often cited as a major impediment to the widespread use of this technology (although it should be remembered that a different load combination should be used for the calculation of the structural capacity under normal conditions and under accidental fire conditions). However, little attention is paid to the need for adequate protection of composites against vandalism, which can occur much more frequently and cause more sudden destruction than fire. The long-term durability of FRP prestressing systems is not entirely understood yet—further extensive research is needed to investigate both all the rheological phenomena in the FRPs and possible galvanic corrosion between carbon fibres and steel anchor hardware. It seems that some of the prestressing systems for FRP strips currently being offered on the market underestimate the importance of galvanic corrosion risk. Therefore, research is needed that will lead to an effective modification of anchoring systems; one that will guarantee the required durability.

The co-operation of stiff and flexible adhesive layers in the strengthening of concrete structures using FRP composite may eliminate the disadvantage of brittle and rapid failure of the concrete–FRP composite joint. The presented unique tests indicated that the ductility of the repaired structure increased significantly, improving safety reserve, even for the severely damaged beam. Parallel bonding of CFRP laminates on stiff and flexible adhesive layers increases the work, which must be performed by external forces to damage the strengthened structure, whereas the additional cost of the proposed flexible protection is relatively low. The offered strengthening solution, using the parallel application of stiff and flexible adhesive layers, can be applied in repair and strengthening of severely damaged structures, when it is necessary to ensure the safety of the structure, for instance, for the time needed to evacuate users.

Certainly, the present study showed that the use of an FRP strip on a highly flexible polymer may improve the safety of the structure. The authors are aware that it is difficult to draw general, generalised conclusions from the test performed on only one element. At the current stage, the research shows only a global qualitative effect of the presented solution and not a quantitative one.

Therefore, further research in this area is highly desirable to confirm or deny whether polymers of low stiffness and high deformability (PXBM) can be considered as structural materials equivalent to commonly used adhesive resins. These studies should also take into account the influence of long-term rheological effects occurring in the adhesive layer. Another important aspect for further consideration is the possibility of replacing carbon fibre tape (CFRP) with a composite made from cheaper and more accessible fibres.

## Figures and Tables

**Table 1:** The basic properties of composite materials.

Material Property	GFRP	BFRP	AFRP	CFRP
Density [kg/m^3^]	2100	2600	1300	1650
Tensile Strength [MPa]	1000–1500	850–1550	1500–2500	1200–3700
Tensile Elastic Modulus [GPa]	40–50	30–90	40–120	120–580
Thermal Conductivity [W/m·K]	1	0.02	0.8	1.4
Coefficient of Linear Thermal Expansion [1/K]	10^−5^	4 × 10^−6^	5 × 10^−6^	0.5 × 10^−6^

**Table 2:** The rheological properties of composite materials [26].

Material Property	GFRP	AFRP	CFRP
Strain due to Creep [‰]	3.0–10.0	1.5–10.0	<0.1
Relaxation [%]	1.8–2.0	5.0–10.0	0.5–1.0
Long-term Tensile Strength	(0.4 ÷ 0.7) f_Lu_	(0.5 ÷ 0.7) f_Lu_	>0.9 f_Lu_

**Table 3:** The basic material properties of epoxy resins and polyurethane adhesives.

Material	Type	Tensile Strength [MPa]	Deformability Limit [%]	Tensile Elastic Modulus [MPa]	Density [kg/m^3^]
Sikadur-30	Epoxy Resins	20–29	0.3	11,200	1600
Sikadur-330		30	0.9	4500	1330
PT	Polyurethane Adhesives	20	15	700	1080
PS		2.5	40	18	1450
PM		1.4	110	4	970
PSM		2.2	80	6	860

**Table 4:** The tensile elastic modulus (MPa) of the adhesives in a 20–100 °C range [28].

Temperature [°C]	20	40	60	80	100
Sikadur-30	9410	7410	942	107	85
Sikadur-330	3760	3400	194	62	46
PT	541	519	499	478	454
PS	19.80	19.23	18.62	17.33	16.29
PM	2.97	2.86	2.75	2.65	2.55
PSM	4.43	4.24	4.07	3.91	3.89

## Data Availability

The data presented in this study are available on request from the corresponding author. The data are not publicly available due to privacy reason.
